# Supplementary material for: Evolution of a Human-Specific De Novo Open Reading Frame and Its Linked Transcriptional Silencer
Source: Int J Mol Sci. 2024 Mar 31;25(7):3924. doi: 10.3390/ijms25073924 (PMC11011693; doi:10.3390/ijms25073924)
Supplement: Supplementary file 1 [file ijms-25-03924-s001.zip › ijms-2914624-supplementary.pdf]

Evolution of a human specific *de novo* open reading frame and its linked transcriptional silencer

Nicholas Delihas

https://orcid.org/0000-0002-1704-2587

Department of Microbiology and Immunology
Renaissance School of Medicine
Stony Brook University
Stony Brook, NY 11794 USA

Email: Nicholas.delihas@stonybrook.edu
Tel. # 001 631 286-9427

Supplementary file S1

Figure S1a. Predicted transmembrane domain of the 68 aa sequence. Data obtained using the DeepTMHMM prediction program (https://dtu.biolib.com/DeepTMHMM) [1].

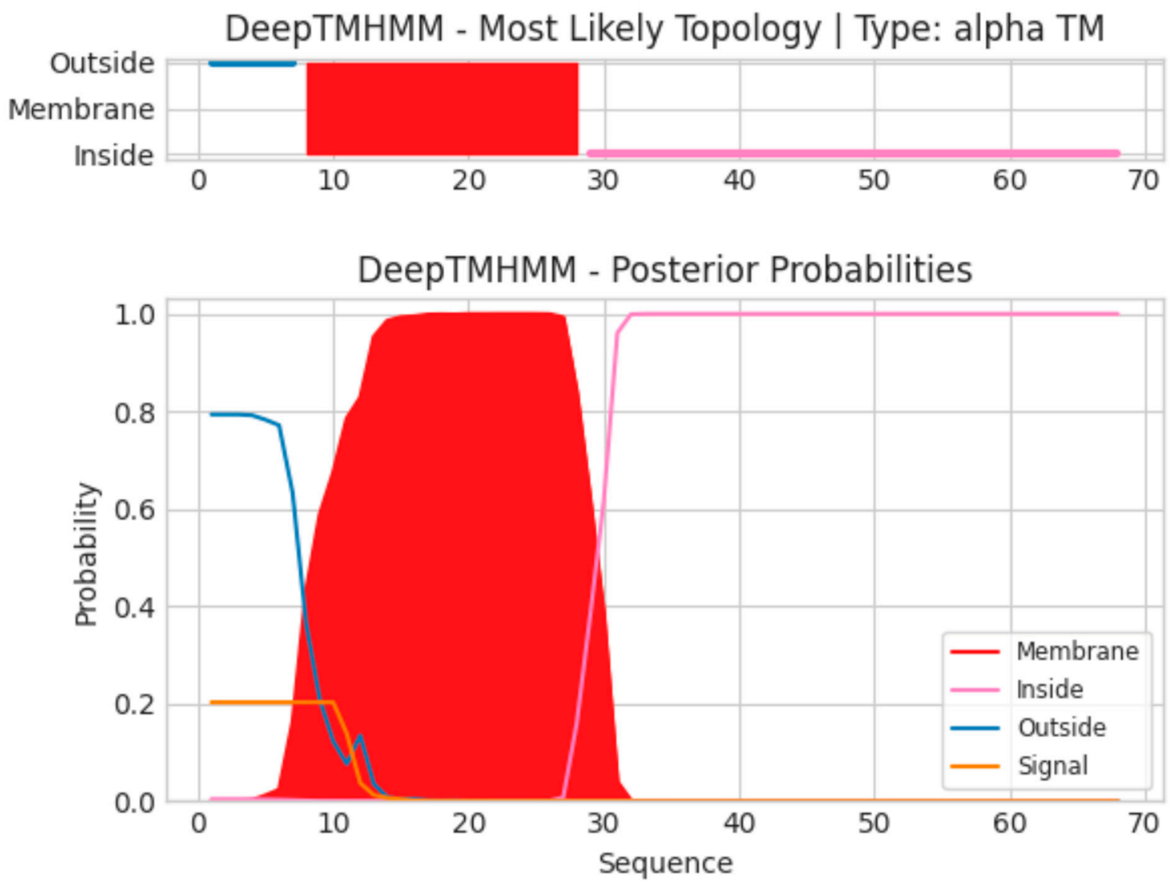

Figure S1b. The 68 aa ORF base composition. The 68 aa ORF nt sequence is rich in G+C.

A (15% 33) | T (22% 43) | G (31% 66) | C (32% 69)

Figure S2. Alignment of nt sequences from the 68 aa ORF, the intervening sequence and LOC130067579 ATAC-STARR-seq. lymphoblastoid silencer. Color code; teal: 3'end of 68 aa ORF and 5' end of the silencer sequence LOC130067579 that overlap; green: the silencer sequence LOC130067579 that overlaps the intervening sequence, purple: the start or the 107 aa sequence: All overlapping alignments show 100% identity.

```
CLUSTAL O(1.2.4) multiple sequence alignment

68aa.3'end.positions51-207                         ggaccgcgacctccgcaagacgctaatggtgcgcgacaacctggccttcggcggcccgga   60
Intervening.sequence                               ------------------------------------------------------------   0
LOC130067579.ATAC-STARR-seq.lymphoblastoid.silent  -----------------------------tgcgcgacaacctggccttcggcggcccgga   31

68aa.3'end.positions51-207                         ggtctga-----------------------------------------------------   67
Intervening.sequence                               -------gccgacttgcaaaggggataggcgggcggcaccgggcgccctcccccagcccg   53
LOC130067579.ATAC-STARR-seq.lymphoblastoid.silent  ggtctgagccgacttgcaaaggggataggcgggcggcaccgggcgccctcccccagcccg   91

68aa.3'end.positions51-207                         ------------------------------------------------------------   67
Intervening.sequence                               ccccgcccgcccagcccggagacccccaaggcagagggaggccggcctgttggccctcca   113
LOC130067579.ATAC-STARR-seq.lymphoblastoid.silent  ccccgcccgcccagcccggagacccccaaggcagagggaggccggcctgttggccctcca   151

68aa.3'end.positions51-207                         ------------------------------------------------------------   67
Intervening.sequence                               cgctatccctctgcagcctgggccctcccgacagaggccccaggtgcgctggcagtggag   173
LOC130067579.ATAC-STARR-seq.lymphoblastoid.silent  cgctatccctctgcagcctgggccctcccgacagaggccccaggtgcgctggcagtggag   211

68aa.3'end.positions51-207                         ------------------------------------------------------------   67
Intervening.sequence                               gtggggcacttaggtgcctggctggcccagggcttgctctccgtgtcaagccgactcacc   233
LOC130067579.ATAC-STARR-seq.lymphoblastoid.silent  gtggggcacttaggtgcct-----------------------------------------   230

68aa.3'end.positions51-207                         ------------------------------------------------------------   67
Intervening.sequence                               cagagcccaccctcccaagctcaggggcatcctccgctgggccccagtgcctttgcgctg   293
LOC130067579.ATAC-STARR-seq.lymphoblastoid.silent  ------------------------------------------------------------   230

68aa.3'end.positions51-207                         ------------------------------- 67
Intervening.sequence                               cgcagcactctgccctccactggactcaggc 324
LOC130067579.ATAC-STARR-seq.lymphoblastoid.silent  ------------------------------- 230
```

230 bp, length of silencer

38 bp, length overlap of LOC130067579.ATAC-STARR-seq. with 68aa ORF nt sequence

192 bp, length of LOC130067579.ATAC-STARR-seq. overlap with intervening sequence

132 bp, length of intervening sequence between the silencer and 107 aa ORF with no overlap

Figure S3a. Alignment of the *SMIM45* 68 aa elephant shark nt sequence, the 68aa nt. (207) human sequence, LOC130067579 ATAC-STARR-seq. lymphoblastoid silencer, and the ATAC-STARR-seq. silencer that overlaps the 68aa ORF. The alignment shows

co-alignment of the total silencer sequence and the silencer that overlaps the 68 aa ORF with the start of the 68 aa ORF sequence (yellow highlighted). Therefore, the alignment shows no synteny as the silencer sequence that overlaps the 68 aa ORF sequence should align at the end of the 68 aa ORF up to position 207. The alignment thus appears to be random.

```
CLUSTAL O(1.2.4) multiple sequence alignment


SMIM45.68aa.elephant.shark                              ATGCCTCACTTCCTGGACTGGTTTGTGCCCGTCTACCTGTTGGCGTCCATCCTGGTCCTG 60
68aa.nt.(207)nt.human                                   atgccgcacttcctggactggttcgtgccggtctacttggtcatctcggtcctcattctg 60
LOC130067579.ATAC-STARR-seq.lymphoblastoid.silent.ref   -tgcgacaacctggccttcggcggcccggaggtctgagccgacttgcaaaggggatag 59
ATAC-STARR-seq.in.68aa.ORF                              -tgcgacaacctggccttcggcggcccggaggtctga--------------------- 38
                                                         ***   **  ***** **     *  ** *    *

SMIM45.68aa.elephant.shark                              GTGG----CATTCGGCGCCTGCGTTTACTACTGCGAGCCCGGGCTAC-AAGACGCCCACA 115
68aa.nt.(207)nt.human                                   gtggg----cttcggcgcctgcatctactacttcgagccgggcctgc-aggaggcgcaca 115
LOC130067579.ATAC-STARR-seq.lymphoblastoid.silent.ref   gcgggcggcaccgggcgccctcccccagcccgccccgcccgcccagcccggagaccccca 119
ATAC-STARR-seq.in.68aa.ORF                              ------------------------------------------------------------ 38

SMIM45.68aa.elephant.shark                              AGTGGCGGACTCAGGGTCCCGTCGGCGAACAGGACGTC---------------------- 153
68aa.nt.(207)nt.human                                   agtggcgcatgcagcgcccctggtggaccgcgacctc----------------------- 153
LOC130067579.ATAC-STARR-seq.lymphoblastoid.silent.ref   --aggcagagggaggccggcctgttggccctccacgctatccctctgcagcctgggccct 177
ATAC-STARR-seq.in.68aa.ORF                              ------------------------------------------------------------ 38

SMIM45.68aa.elephant.shark                              --CGCAAGGCGTCGATGGTCCGGGAGAACATGGGATTCCGTCCCCCCGACCTCTGA 207
68aa.nt.(207)nt.human                                   --cgcaagacgctaatggtgcgcgacaacctggccttcggcggcccggaggtctga 207
LOC130067579.ATAC-STARR-seq.lymphoblastoid.silent.ref   cccgacagaggccccaggtgcgctggcagtggaggtggggcact-taggtgcct-- 230
ATAC-STARR-seq.in.68aa.ORF                              -------------------------------------------------------- 38
```

Figure S3b. Alignment of the silencer that overlaps the intervening sequence**,** the LOC130067579 ATAC-STARR-seq. lymphoblastoid silencer sequence, the human 68 aa ORF sequence, and the redfish *SMIM45* (Ensembl primaryassembly:fErpCal1.1:12:30561086:30562492:-1) sequence. The alignment shows co-alignment of the silencer with the 5' end of the 68 aa ORF thus displaying no synteny and random alignments.

```
silencer.overlap.with.intervening.only.human                  ------------------------------------------------------------  0
LOC130067579.ATAC-STARR-seq.lymphoblastoid.silent.ref         ------------------------------------------------------------  0
68aa.nt.(207)nt.human                                         ------------------------------------------------------------  0
red.fish.primary_assembly:fErpCal1.1:12:30561086:30562492:-1  tgtacaatgttagataaatcaaggaagtaatgtgtttcttatccttccacagcccccacc  600

silencer.overlap.with.intervening.only.human                  --------------------------------------gccgacttgcaaaggggatag  21
LOC130067579.ATAC-STARR-seq.lymphoblastoid.silent.ref         -tgcgcgacaacctggccttcggcggcccggaggtctgagccgacttgcaaaggggatag  59
68aa.nt.(207)nt.human                                         atgccgcacttcctggactggttcgtgccggtctacttggtcatctcggtcctc----at  56
red.fish.primary_assembly:fErpCal1.1:12:30561086:30562492:-1  atgccgcacttcttagactggtttgtccctgtgtatttgatgatctctattcta----at  656
                                                                                                          **            *

silencer.overlap.with.intervening.only.human                  gcgggcggcaccgggcgccctcccccagcccgccccgcccgcccagcccggagacccc-c  80
LOC130067579.ATAC-STARR-seq.lymphoblastoid.silent.ref         gcgggcggcaccgggcgccctcccccagcccgccccgcccgcccagcccggagacccc-c  118
68aa.nt.(207)nt.human                                         tctggtggcttcggcgcctgcatctactacttcgagccgggcctgcaggaggcgcacaa  116
red.fish.primary_assembly:fErpCal1.1:12:30561086:30562492:-1  tctggttggatttggagcctgcatctactacttcgaacccggcctacaagaagcccacaa  716
                                                               * **  *     ** ***  *  * *   *  *   ** * **  *  *  *  * *

silencer.overlap.with.intervening.only.human                  aaggcagagggaggccggcctgttggccctccacgctatccct---------ctgcag--  129
LOC130067579.ATAC-STARR-seq.lymphoblastoid.silent.ref         aaggcagagggaggccggcctgttggccctccacgctatccct---------ctgcag--  167
68aa.nt.(207)nt.human                                         gtggcgcatgcagcgcccccctggtggaccgcgacctccgcaagacgctaatggtgcgcga  176
red.fish.primary_assembly:fErpCal1.1:12:30561086:30562492:-1  gtggcggacacagaaacctctggttgaaagagagactaacaaagttttaatgatgcagga  776
                                                                ***  *   **      *** * *      *      *             ***

silencer.overlap.with.intervening.only.human                  ---c------ctgggccctcccgaca-------------gaggccccaggtgcgctggca  167
LOC130067579.ATAC-STARR-seq.lymphoblastoid.silent.ref         ---c------ctgggccctcccgaca-------------gaggccccaggtgcgctggca  205
68aa.nt.(207)nt.human                                         caacctggccttcggcggcccggaggtctga-----------------------------  207
red.fish.primary_assembly:fErpCal1.1:12:30561086:30562492:-1  caatttggatttccgtgctccggatgtatgacagcaatttgggaggctgcttgccttgtc  836
                                                                         *  *    ** **

silencer.overlap.with.intervening.only.human                  gtggaggtggggcacttaggtgcct-----------------------------------  192
LOC130067579.ATAC-STARR-seq.lymphoblastoid.silent.ref         gtggaggtggggcacttaggtgcct-----------------------------------  230
68aa.nt.(207)nt.human                                         ------------------------------------------------------------  207
red.fish.primary_assembly:fErpCal1.1:12:30561086:30562492:-1  cttgagctcaacagcatctgaaaattcttttcttttcttttctcagttcccccacatcta  896
```

Figure S4a. Alignment of *Xenopus tropicalis* (tropical clawed frog sequence that is homologous to the silencer1 segement in the 68 aa ORF (exonic silencer).

```
#=======================================
#
# Aligned_sequences: 2
# 1: Xenopus.homolo.t.silencer.in.68aa
# 2: silencer.in.68aa.38bp
# Matrix: EDNAFULL
# Gap_penalty: 10.0
# Extend_penalty: 0.5
#
# Length: 39
# Identity:      27/39 (69.2%)
# Similarity:    27/39 (69.2%)
# Gaps:           2/39 ( 5.1%)
# Score: 85.0
#
#
#=======================================

Xenopus.homol      1 tgagggacaatctggcctacagcctccctgatgtatag-     38
                     ||.|.|||||.|||||||.|.||..|||.||.||.| |
silencer.in.6      1 tgcgcgacaacctggccttcggcggcccggaggtct-ga     38


#---------------------------------------
#---------------------------------------
```

Figure S4b. Alignment of *Caretta caretta* (Loggerhead turtle) sequence that is homologous to the silencer1 nt sequence within the 68 aa ORF.

```
#=======================================
#
# Aligned_sequences: 2
# 1: turtle.homolog.to.silencer.in.68aa
# 2: silencer.in.68aa.38bp
# Matrix: EBLOSUM62
# Gap_penalty: 10.0
# Extend_penalty: 0.5
#
# Length: 38
# Identity:      27/38 (71.1%)
# Similarity:    27/38 (71.1%)
# Gaps:           0/38 ( 0.0%)
# Score: 155.0
#
#
#=======================================

turtle.homolog     1 tacgggataaccttgcctttggggtgcctgaggtctag     38
                     |.||.||.|||||.|||||.||.|..||.||||||||..
silencer.in.66aa   1 TGCGCGACAACCTGGCCTTCGGCGGCCCGGAGGTCTGA     38


#---------------------------------------
#---------------------------------------
```

.

Figure S5a. Alignment of three sequences: the cape elephant shrew 3’ end of the 68 aa sequence with addition of 40 bps; the 68 aa 38 bp terminal end; and the complete silencer sequence. No significant sequence identity with silencer LOC130067579 is

found in the 40 bp region that extends40. bp beyond the end of the 68 aa ORF sequence of the cape elephant shrew.

```
CLUSTAL O(1.2.4) multiple sequence alignment


cape.elephant.shrew.3'end.68aa.+40bp      tgcgtgacaacctggccttcggaggccccgaggtctgagcgggctgatggcgctcgctgc 60
68aa.38.bp.3'end                          TGCGCGACAACCTGGCCTTCGGCGGCCCGGAGGTCTGA---------------------- 38
ATAC-STARR-seq.lymphoblastoid.silent      tgcgcgacaacctggccttcggcggcccggaggtctgagccgacttgcaaaggggatagg 60
                                          **** ***************** ***** *********

cape.elephant.shrew.3'end.68aa.+40bp      gccctg---------------ccccttc-------------------tggagaccctccc 86
68aa.38.bp.3'end                          ------------------------------------------------------------ 38
ATAC-STARR-seq.lymphoblastoid.silent      cgggcggcaccgggcgccctcccccagcccgccccgcccgcccagcccggagacccccaa 120

cape.elephant.shrew.3'end.68aa.+40bp      ------------------------------------------------------------ 86
68aa.38.bp.3'end                          ------------------------------------------------------------ 38
ATAC-STARR-seq.lymphoblastoid.silent      ggcagagggaggccggcctgttggccctccacgctatccctctgcagcctgggccctccc 180

cape.elephant.shrew.3'end.68aa.+40bp      -------------------------------------------------- 86
68aa.38.bp.3'end                          -------------------------------------------------- 38
ATAC-STARR-seq.lymphoblastoid.silent      gacagaggccccaggtgcgctggcagtggaggtggggcacttaggtgcct 230
```

Figure S5b. Alignment of the cape golden mole sequence that is homologous to silencer2 that overlaps the intervening sequence. Alignment obtained by using the Blast align 2 sequences (https://blast.ncbi.nlm.nih.gov/Blast.cgi?PROGRAM=blastn&PAGE_TYPE=BlastSearch&LINK_LOC=blasthome).

```
>cape.golden.mole.homolog.silencer
ccctgttcct gccttgcctg aagaccctcc gaggagaggg aagacgaccc cctgatcctc
      61 cacgctatcc ctctgcagcc

>silencer.overlap.with.intervening.only.human
gccgacttgc aaaggggata ggcgggcggc accgggcgcc ctcccccagc ccgccccgcc
      61 cgcccagccc ggagaccccc aaggcagagg gaggccggcc tgttggccct ccacgctatc
     121 cctctgcagc ctgggccctc ccgacagagg ccccaggtgc gctggcagtg gaggtggggc
     181 acttaggtgc ct
```

Job Title
cape.golden.mole.homolog.silencer

Query Descr
cape.golden.mole.homolog.silencer
Query Length
80
Subject ID
lcl|Query_7326325 (dna)
Subject Descr
silencer.overlap.with.intervening.only.human
Subject Length
192

silencer.overlap.with.intervening.only.human Sequence ID: **Query_7326325**Length: **192**Number of Matches: **1**

Range 1: 59 to 131 Graphics
Next Match
Previous Match

Alignment statistics for match #1

Score	Expect	Identities	Gaps	Strand
55.4 bits(60)	3e-13	58/74(78%)	2/74(2%)	Plus/Plus

```
Query  8     CCTGCCTTGCCTGAAGACCCTCCGAGG-AGAGGGAAGACGACCCCCTGATCCTCCACGCT  66
             || |||  ||| | |||||| || ||| ||||| | || ||   ||  ||||||||||
Sbjct  59    CCCGCCCAGCCCGGAGACCC-CCAAGGCAGAGGGAGGCCGGCCTGTTGGCCCTCCACGCT  117

Query  67    ATCCCTCTGCAGCC  80
             ||||||||||||||
Sbjct  118   ATCCCTCTGCAGCC  131
```

Figure S6. Evolutionary relationships of the Afrothere. Drawing modified from [2], with permission.

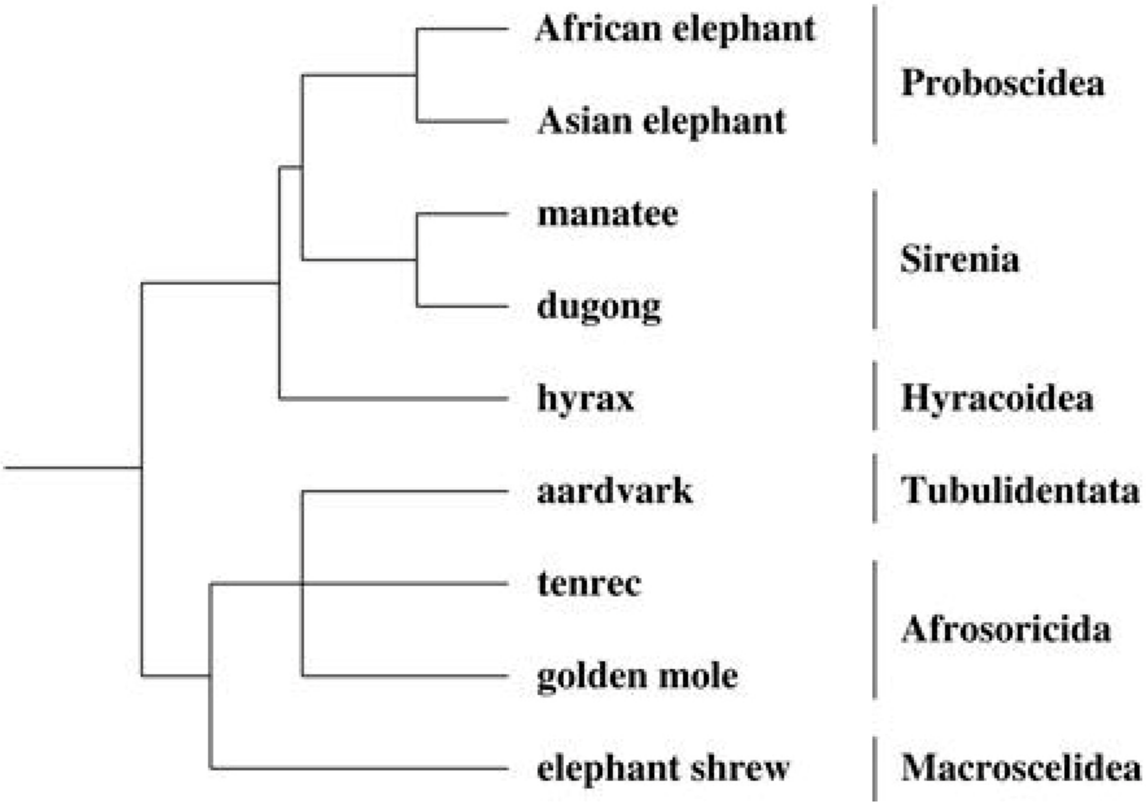

Figure S7 a. Alignment of the elephant nt sequence homologous to the early development sequence with the analogous human early development nt sequence. Data obtained using Nucleotide BLAST two sequences (https://blast.ncbi.nlm.nih.gov/BlastAlign.cgi).

>elephant.homolog.to.107aa.nt.190-243.bp
tcaggtttagaatgggtgaggtgggtgtggctgggggtgtgcagaagggcaagccc

>human107aa.nt.fragment.190-243.bp
tcaggcttggaactggtgagggtgtgtggtgggggtatgcagagggataagacc

Job Title

elephant.homolog.to.107aa.nt.190-243.bp

Query Descr

elephant.homolog.to.107aa.nt.190-243.bp

Query Length

56

Subject Descr

human107aa.nt.fragment.190-243.bp

Subject Length

54

human107aa

Sequence ID: Query_346459 Length: 54 Number of Matches: 1

Range 1: 1 to 54 Graphics

Next Match

Previous Match

Alignment statistics for match #1

Score	Expect	Identities	Gaps	Strand
41.0 bits(44)	1e-09	44/56(79%)	2/56(3%)	Plus/Plus

```
Query  1    TCAGGTTTAGAATGGGTGAGGTGGGTGTGGCTGGGGGTGTGCAGAAGGGCAAGCCC  56
            ||||| || |||  ||||||| | |||||| ||||||| |||||| ||  ||| ||
Sbjct  1    TCAGGCTTGGAACTGGTGAGG-GTGTGTGG-TGGGGGTATGCAGAGGGATAAGACC  54
```

Figure S7 b. The elephant aa sequence homologous to SGLELVRVCGGGMQRDKT aligned with the human early developmental sequence. Point mutations and a frameshift mutations alter the early developmental aa sequence.

>elephant.Frame1.homolog
SGLEWVRWVWLGVCRRAS

>human.SGLELVRVCGGGMQRDKT.nt.seq
SGLELVRVCGGGMQRDKT

Job Title
elephant.Frame1.homolog
Query Descr
elephant.Frame1.homolog
Query Length
18
Subject Descr
human.107aa.SGLELVRVCGGGMQRDKT.nt.seq
Subject Length
18

human.SGLELVRVCGGGMQRDKT.nt.seq Sequence ID: **Query_501325**Length: **18**Number of Matches: **1**

Range 1: 1 to 9 Graphics
Next Match
Previous Match

Alignment statistics for match #1

Score	Expect	Identities	Positives	Gaps
19.7 bits(39)	8e-05	8/14(57%)	8/14(57%)	5/14(35%)

```
Query  1   SGLEWVRWVWLGVC  14
           SGLE VR     VC
Sbjct  1   SGLELVR-----VC  9
```

Figure S8 a. Alignment of the aardvark nt sequence that is homologous to the early development nt sequence with the analogous human early development nt sequence.

```
#
#
#  Percent Identity  Matrix - created by Clustal2.1
#
#

     1: aardvark.homolog.to.SGLELVRVCGGGMQRDKT.nt.seq  100.00   56.60
     2: SGLELVRVCGGGMQRDKT.nt.seq                        56.60  100.00


CLUSTAL O(1.2.4) multiple sequence alignment


aardvark.homolog.to.SGLELVRVCGGGMQRDKT.nt.seq      tcaggtttagaattggtgagagaggtgtggctggcccagtgtgaagggcaaccc-  54
SGLELVRVCGGGMQRDKT.nt.seq                          tcaggcttggaactggtgagggtgtgtggtgggg-gtatgcagagggataagacc  54
                                                   ***** ** *** ******* * *    *   **   *    ** **  **  *

#========================================
```

No significant E value

Figure S8 b. The aardvark aa sequence homologous to SGLELVRVCGGGMQRDKT aligned with the human early developmental sequence.

>aardvark5'3'Frame.1
SGLELVREVWLAQCEGQP

>SGLELVRVCGGGMQRDKT.nt.seq
SGLELVRVCGGGMQRDKT

Job Title
aardvark5'3'Frame.1
Query Descr
aardvark5'3'Frame.1
Query Length
18
Subject ID
lcl|Query_50429 (amino acid)
Subject Descr
SGLELVRVCGGGMQRDKT.nt.seq
Subject Length
18

SGLELVRVCGGGMQRDKT.nt.seq

Sequence ID: Query_50429 **Length: 18** **Number of Matches: 1**

Range 1: 1 to 7 Graphics

Next Match
Previous Match

Alignment statistics for match #1

Score	Expect	Identities	Positives	Gaps
24.0 bits(49)	2e-06	7/7(100%)	7/7(100%)	0/7(0%)

```
Query  1  SGLELVR  7
          SGLELVR
Sbjct  1  SGLELVR  7
```

Figure S9. a. Alignment of the Lesser hedgehog (tenrec) nt sequence that is homologous to the early development sequence with the analogous human early development nt sequence. **a.**

```
>hedgehog.homolog.early.dev.seq.
ggcagtttagaactggccaggggctgggggtggagggtgcaggagagcagtccc   660

>SGLELVRVCGGGMQRDKT.nt.seq

tcaggcttggaactggtgagggtgtgtggtgggggtatgcagagggataagacc


#
#
#  Percent Identity  Matrix - created by Clustal2.1
#
#

     1: hedgehog.homolog.early.dev.seq.  100.00   55.56
     2: SGLELVRVCGGGMQRDKT.nt.seq         55.56  100.00

CLUSTAL O(1.2.4) multiple sequence alignment


hedgehog.homolog.early.dev.seq.      ggcagtttagaactggccaggggctgggggtggagggtgcaggagagcagtccc 54
SGLELVRVCGGGMQRDKT.nt.seq            tcaggcttggaactggtgagggtgtgtggtgggggtatgcagagggataagacc 54
                                         * ** *******  ****  ** **  ** *  *****  *   *   **
```

No significant E value

Figure S9. b. The Lesser hedgehog aa sequence homologous to SGLELVRVCGGGMQRDKT aligned with the human early developmental sequence.

```
#=======================================
#
# Aligned_sequences: 2
# 1: hedgehog.5'3'Frame1
# 2: SGLELVRVCGGGMQRDKT.
# Matrix: EBLOSUM62
# Gap_penalty: 10.0
# Extend_penalty: 0.5
#
# Length: 18
# Identity:       6/18 (33.3%)
# Similarity:     7/18 (38.9%)
# Gaps:           0/18 ( 0.0%)
# Score: 20.0
#
#
```

```
>hedgehog.5'3'Frame1
GSLELARGWGWRVQESSP

>SGLELVRVCGGGMQRDKT.
SGLELVRVCGGGMQRDKT


hedgehog.5'       1 GSLELARGWGWRVQESSP     18
                    ..|||.|..|..:|....
SGLELVRVCGGGM     1 SGLELVRVCGGGMQRDKT     18

#---------------------------------------
#---------------------------------------
```

No significant E value.

Figure S10. a. Alignment of the cape golden mole nt sequence that is homologous to the early development sequence with the analogous human early development nt sequence.

a.

```
>cape.golden.mole.homolog
ttagaactggcaagg-----ggtgtatggctggga
tgtgcgcaagggcaaggtc


>SGLELVRVCGGGMQRDKT.nt.seq
tcaggcttggaactggtgagggtgtgtggtgggggtatgcagagggataagacc
```

```
#=======================================
#
# Aligned_sequences: 2
# 1: cape.golden.mole.homolog
# 2: SGLELVRVCGGGMQRDKT.nt.seq
# Matrix: EDNAFULL
# Gap_penalty: 10.0
# Extend_penalty: 0.5
#
# Length: 55
# Identity:      33/55 (60.0%)
# Similarity:    33/55 (60.0%)
# Gaps:           7/55 (12.7%)
# Score: 95.0
#
#
#=======================================

cape.golden.m      1 ------ttagaactggcaaggggtgtatggctgggatgtgcgcaagggca     44
                           ||.|||||||..| ||||||.|||..|||.|.|||..|.||..|
SGLELVRVCGGGM      1 tcaggcttggaactggtga-gggtgtgtggtgggggtatgcagagggata     49

cape.golden.m     45 aggtc     49
                     ||..|
SGLELVRVCGGGM     50 agacc     54
```

No significant E value

Figure S10 b. The cape golden mole aa sequence homologous to SGLELVRVCGGGMQRDKT aligned with the human early developmental sequence.

>cape.golden.mole.5'3'Frame1
LELARGVWLGCAQGQG

>SGLELVRVCGGGMQRDKT.
SGLELVRVCGGGMQRDKT

```
#=======================================
#
# Aligned_sequences: 2
# 1: cape.golden.mole.5'3'Frame1
# 2: SGLELVRVCGGGMQRDKT.
# Matrix: EBLOSUM62
# Gap_penalty: 10.0
# Extend_penalty: 0.5
#
# Length: 22
# Identity:       6/22 (27.3%)
# Similarity:     6/22 (27.3%)
# Gaps:          10/22 (45.5%)
# Score: 14.5
#
#
#=======================================

cape.golden.m      1 --LELARGVWLGCAQGQG----      16
                       |||.|    .|..|..
SGLELVRVCGGGM      1 SGLELVR----VCGGGMQRDKT      18
```

No significant ER value.

Figure S11 a. Alignment of the human 107 aa mRNA, the early developmental sequence, the cape elephant shrew genomic nt sequence, and the 68 aa nt sequence that is homologous to the early development sequence with the analogous human early development nt sequence. The alignment shows no synteny between the 68 aa ORF and 107 aa ORF nt sequences. However, the human SGLELVRVCGGGMQRDKT nt sequence aligns with a segments of the Cape elephant shrew, starting with position 33353. This Cape elephant shrew sequence was used in Figs. S11 b,c.

```
human.107aa.mRNA                        tttggagggatgtcagcttggccagcccagccgcaccccgcggggc-----tcaggctt  197
SGLELVRVCGGGMQRDKT.nt.seq               ----------------------------------------------------tcaggctt  8
Septin3-CENPM.cape.eleph.shrew.revcompl tgaggtggaacttgtgcagagttcagtccagctccaccccttcagagggcagtgaggaag  33360
68aa.nt.(207)nt.human                   ------------------------------------------------------------  207
                                                                                               |
human.107aa.mRNA                        ----ggaactggtgagggtgtgtggtggg--ggtatgcagagg-----------------  234
SGLELVRVCGGGMQRDKT.nt.seq               ----ggaactggtgagggtgtgtggtggg--ggtatgcagagg-----------------  45
Septin3-CENPM.cape.eleph.shrew.revcompl ggcagaccctaatgggaaggtgaggtgcatgtgtttgcatctgagtgcatgagtggagat  33420
68aa.nt.(207)nt.human                   ------------------------------------------------------------  207
```

Figure S11 b. The nt sequence alignment of the cape elephant shrew sequence that aligns with early developmental sequence (from Figure S11a) and the the early developmental sequence. The results show an insignificant identity of 38%.

>cape.eleph.shrew.homolog
tgaggaagggcagaccctaatgggaaggtgaggtgcatgtgtttgcatctg

>SGLELVRVCGGGMQRDKT.nt.seq
tcaggcttggaactggtgagggtgtgtggtggggtatgcagagggataagacc

```
#=======================================
#
# Aligned_sequences: 2
# 1: cape.eleph.shrew.homolog
# 2: SGLELVRVCGGGMQRDKT.nt.seq
# Matrix: EDNAFULL
# Gap_penalty: 10.0
# Extend_penalty: 0.5
#
# Length: 73
# Identity:      28/73 (38.4%)
# Similarity:    28/73 (38.4%)
# Gaps:          41/73 (56.2%)
# Score: 57.0
#
#
#=======================================

cape.eleph.sh      1 tgaggaagggcagaccctaatgggaa--ggtga-ggtgcatgt-------     40
                              .|||.|     |.||||  ||||| ||||  |||
SGLELVRVCGGGM      1 ---------tcaggc-----ttggaactggtgagggtg--tgtggtgggg     34

cape.eleph.sh     41 gtttgcatctg------------     51
                     ||.||||   |
SGLELVRVCGGGM     35 gtatgca---gagggataagacc     54


#---------------------------------------
```

Figure S11. c. The aa sequence alignment of the homologous cape elephant shrew sequence with the early developmental sequence. The results show an insignificant identity of 17%.

```
>cape.eleph.shrew.Frame2
EEGQTLMGR-GACVC

>SGLELVRVCGGGMQRDKT.nt.seq
SGLELVRVCGGGMQRDKT

#=======================================
#
# Aligned_sequences: 2
# 1: cape.eleph.shrew.Frame2
# 2: SGLELVRVCGGGMQRDKT.nt.seq
# Matrix: EBLOSUM62
# Gap_penalty: 10.0
# Extend_penalty: 0.5
#
# Length: 23
# Identity:       4/23 (17.4%)
# Similarity:     5/23 (21.7%)
# Gaps:          14/23 (60.9%)
# Score: 7.5
#
#
#=======================================

cape.eleph.sh      1 EEGQTLMGRGACVC---------     14
                      .|..|:.    ||
SGLELVRVCGGGM      1 -SGLELVR----VCGGGMQRDKT     18

#---------------------------------------
```

Figure S12. Displayed are the alignments with color coordinated DNA bases of the early developmental sequence and homologous sequences from genomes of the Afrothere, mouse and human species. Data was obtained using MAFFT online service: multiple sequence alignment, interactive sequence choice and visualization, http://msa.biojs.net/ [3]. The totally conserved bases are readily visible. The first six

bases of the cape golden mole sequence are missing that indicates no significant alignment in the region. Thus, the homologous sequence is smaller in the cape golden mole. This may suggest a closer proximity to the root species than the other species.

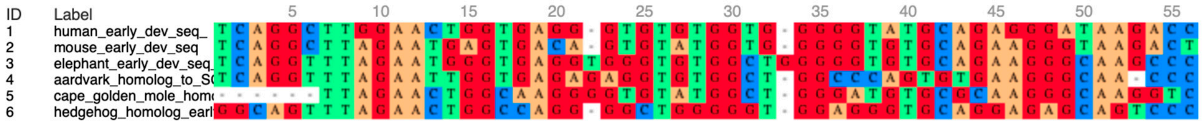

References

1. Hallgren J. et al. DeepTMHMM predicts alpha and beta transmembrane proteins using deep neural networks. bioRxiv preprint 2022 https://doi.org/10.1101/2022.04.08.487609

2. . Nishihara H, Satta Y, Nikaido M, Thewissen JG, Stanhope MJ, Okada N. A retroposon analysis of Afrotherian phylogeny.
Mol Biol Evol. 2005, 22(9):1823-1833. https://doi: 10.1093/molbev/msi179.

3. Katoh K, Rozewicki J, Yamada KD, MAFFT online service: multiple sequence alignment, interactive sequence choice and visualization. Brief Bioinform. 2019 20:1160-1166. https://doi: 10.1093/bib/bbx108
